# Identification of a signalling molecule involved in bacterial intergeneric communication

**DOI:** 10.1099/mic.0.2007/009050-0

**Published:** 2007-10

**Authors:** Hua Xie, Xinghua Lin, Bing-Yan Wang, Jie Wu, Richard J. Lamont

**Affiliations:** 1 School of Dentistry, Meharry Medical College, Nashville, TN 37208, USA; 2 Department of Periodontics and Endodontics, State University of New York at Buffalo, Buffalo, NY 14214, USA; 3 Department of Oral Biology, University of Florida, Gainesville, FL 32610-0424, USA

## Abstract

The development of complex multispecies communities such as biofilms is controlled by interbacterial communication systems. We have previously reported an intergeneric communication between two oral bacteria, *Streptococcus cristatus* and *Porphyromonas gingivalis*, that results in inhibition of *fimA* expression. Here, we demonstrate that a surface protein, arginine deiminase (ArcA), of *S. cristatus* serves as a signal that initiates intergeneric communication. An ArcA-deficient mutant of *S. cristatus* is unable to communicate with *P. gingivalis*. Furthermore, arginase activity is not essential for the communication, and ArcA retains the ability to repress expression of *fimA* in the presence of arginine deiminase inhibitors. These results present a novel mechanism by which intergeneric communication in dental biofilms is accomplished.

## INTRODUCTION

Human dental plaque is a multispecies microbial biofilm that is associated with two common oral diseases, dental caries and periodontal disease. More than 700 bacterial species have been detected in the oral cavity, over 50 % of which are identified by culture-independent molecular techniques (Aas *et al.*, 2005). Formation of dental plaque is a highly organized developmental process involving a specific sequence of colonization that results in spatially and temporally organized structures (Kolenbrander *et al.*, 2006). Formation of dental plaque is initiated by Gram-positive species, including streptococci and *Actinomyces* spp., which recognize salivary receptors exposed on the tooth surfaces (Gibbons *et al.*, 1991; Li *et al.*, 2000; Scannapieco *et al.*, 1995). These early colonizers in turn provide new surfaces that attract and recruit succeeding organisms including Gram-negative potential pathogens, such as *Porphyromonas gingivalis* and *Aggregatibacter* (*Actinobacillus*) *actinomycetemcomitans* (Kolenbrander *et al.*, 2002). Therefore, the early colonizers play a key role in the development of the dental plaque biofilm.

It is recognized that cell–cell communication occurs between bacterial strains, species and genera. A universal language for interspecies bacterial communication is autoinducer-2 (AI-2). LuxS, the AI-2 synthase, has been discovered in many oral bacteria, including *Streptococcus mutans*, *S. oralis*, *S. gordonii*, *P. gingivalis* and *A. actinomycetemcomitans* (Chung *et al.*, 2001; James *et al.*, 2006; Merritt *et al.*, 2005; Rickard *et al.*, 2006). LuxS-dependent intercellular communication appears to play an important role in biofilm formation in the oral cavity. McNab *et al.* (2003) found that a *S. gordonii luxS* mutant was unable to form normal biofilms with a LuxS-deficient strain of *P. gingivalis*, and complementation of the *luxS* mutation in *S. gordonii* restored normal biofilm formation with the *luxS*-deficient *P. gingivalis*. In addition to communication mediated through soluble extracellular signalling molecules, interspecies crosstalk can occur through direct cell-to-cell contact (Aoki *et al.*, 2005). We reported earlier that expression of the *P. gingivalis fimA* gene, encoding the long fimbrial major subunit protein, is repressed by surface extracts of *Streptococcus cristatus* (Xie *et al.*, 2000). As the long fimbriae of *P. gingivalis* are required to initiate heterotypic biofilm formation with oral streptococci, substrata of *S. cristatus* do not support the development of a mixed biofilm with *P. gingivalis* (Xie *et al.*, 2000). We show here that arginine deiminase (ArcA) is the inhibitory molecule of *S. cristatus*. The ability of *S. cristatus* to communicate with *P. gingivalis* is diminished in an *arcA* mutant. We also provide evidence that the ability of ArcA to repress expression of the *fimA* in *P. gingivalis* is not correlated with its enzymic activity. This work presents a novel inter-species contact-dependent communication system between *P. gingivalis* and *S. cristatus*.

## METHODS

### Bacterial strains and growth conditions.

The bacterial strains and plasmids are listed in Table 1. *Streptococcus* strains were grown in Trypticase peptone broth (TPB) supplemented with 0.5 % glucose at 37 °C under aerobic conditions. *S. cristatus* CC5A was used as the parental strain for mutant construction. *P. gingivalis* ATCC 33277 was grown from frozen stocks in Trypticase soy broth (TSB) or on TSB blood agar plates, supplemented with 1 mg yeast extract ml^−1^, 5 μg haemin ml^−1^ and 1 μg menadione ml^−1^, at 37 °C in an anaerobic chamber (85 % N_2_, 10 % H_2_, 5 % CO_2_). *Escherichia coli* DH5*α* was used as the host for plasmids. *E. coli* strains were grown in L broth at 37 °C. Antibiotics were used when appropriate, at the following concentrations: 100 μg gentamicin ml^−1^ for *P. gingivalis*, 200 μg erythromycin ml^−1^ for *E. coli* and 10 μg erythromycin ml^−1^ for *S. cristatus*, 2 μg tetracycline ml^−1^ for *E. coli* and *S. cristatus*, 50 μg ampicillin ml^−1^ and 50 μg kanamycin ml^−1^ for *E. coli*.

### Partial purification of the *S. cristatus* inhibitory protein.

Surface extracts of *S. cristatus* CC5A were collected by sonication and centrifugation (13 000 ***g*** for 30 min) followed by filtration (0.2 μm pore size). The crude extract of CC5A was partially purified by ammonium sulfate fractionation as described earlier (Xie *et al.*, 2004). The fractions precipitated with 33, 42, 50, 55, 60 and 66 % saturated ammonium sulfate were designated AS1, AS2, AS3, AS4, AS5 and AS6, respectively. For further purification, the AS6 fraction (1 ml) was dialysed overnight against Tris buffer (50 mM, pH 7.3). The dialysed sample was then applied to a Blue Sepharose column (GE Healthcare), which was pre-washed with the same Tris buffer (Nelson *et al.*, 2001). The non-bound proteins were collected from the column. Bound proteins were eluted with Tris buffer supplemented with 1 mM NAD^+^.

### Proteomic analysis.

Samples were separated by SDS-PAGE (12 % gel) along with prestained size standards (Bio-Rad). Coomassie-stained protein bands of interest were excised and reduced with 10 μl 45 mM dithiothreitol for 20 min at 37 °C. The gel pieces were then digested with trypsin overnight. The peptides were extracted and reconstituted in 20 μl 0.1 % trifluoroacetic acid. Approximately 0.4 μl of the peptides were spotted onto a MALDI plate. For each individual sample, the MALDI-TOF mass spectrum and the corresponding MS/MS fragmentation spectra were collectively searched against the SWISS-PROT database using GPS Explorer software (Applied Biosystems) running the MASCOT database search engine (Matrix-Science). MALDI-TOF peptide mass maps were internally calibrated to within 20 p.p.m. mass accuracy using trypsin autolytic peptides (*m/z* 842.51 and 2211.10).

### Sequencing of the *S. cristatus arcA* gene.

The entire *arcA* gene of *S. cristatus* CC5A was amplified by the primers 5′-GTACCGATGGTCTTGTTTGA-3′ and 5′-AGGTATTCTAACTCTGCACG-3′, which were designed based on the completely conserved regions among *Streptococus suis flps* (AF546864), *Streptococcus equi* subsp. *zooepidemicus* *arcA* (AB210842) and the *Streptococcus gordonii* DL1 *arc* operon (AF534569). The PCR product was cloned into pCRII-TOPO vector (Invitrogen) and sequenced by using an ABI capillary sequencer (Perkin-Elmer). The sequence is deposited in GenBank (accession number EF435044).

### Construction of the *S. cristatus* *arcA* mutant and *arcA*-complemented strains.

An insertional *arcA* mutant was generated by using ligation-independent cloning of PCR-mediated mutagenesis (LIC-PCR) (Aslanidis & de Jong, 1990). This procedure involved three steps of PCR to introduce a 2.1 kb *ermF*-*ermAM* cassette (Fletcher *et al.*, 1995) into the *arcA* gene. First, the upstream DNA fragment (549 bp) of the *arcA* gene was amplified by using *Taq* RNA polymerase (1 U, Invitrogen) and chromosomal DNA of *S. cristatus* CC5A (0.1 μg) as template with specific primers (5′-ATGTCTACACATCCAATTC-3′ and 5′-GATGTTGCAAATACCGATGAGCATCTGCATACATGTGGTTGA-3′) containing the sequence (underlined) corresponding to the 5′ end of the *ermF*-*ermAM* cassette. The downstream DNA fragment (549 bp) of the *arcA* gene was amplified with specific primers (5′-ACAACGAGGTCCACCACG-3′and 5′-CCTCTAGAGTCGACCTGCAGATCGAAGGTGGAGATGAGTT-3′) containing the sequence (underlined) corresponding to the 3′ end of the *ermF*-*ermAM* cassette. Primers 5′-GCTCATCGGTATTTGCAACA-3′ and 5′-CTGCAGGTCGACTCTAGAGG-3′ were used to amplify the *ermF*-*ermAM* cassette. Each PCR product of the *arcA* gene was then ligated with the *ermF*-*ermAM* cassette by the second PCR step with primers arcAF and ermR or primers ermF and arcAR, respectively. The second-step PCR products (100 ng) were then mixed and used as template with arcAF and arcAR as primers in the third PCR step to create the fragment *arcA-erm-arcA* containing the *ermF*-*ermAM* cassette flanked with upstream and downstream fragments of *arcA*.

The *arcA-erm-arcA* fragment was introduced into *S. cristatus* CC5A cells by DNA transformation (Wang & Kuramitsu, 2005). *arcA*-deficient mutants were constructed via a double-crossover event that introduces the *arcA-erm-arcA* fragment into the CC5A chromosome. The mutants were selected on TPB plates supplemented with erythromycin (10 μg ml^−1^). The mutations were confirmed by PCR analysis, and the one selected for study was designated *S. cristatus* ArcAE.

An *E. coli*–*Streptococcus* shuttle vector was used to construct a complemented strain of ArcAE. To create the *E. coli*–*Streptococcus* shuttle vector, plasmid pSF143 (obtained from L. Tao, University of Illinois, Chicago, IL, USA), which replicates only in *E. coli*, was digested with *Hin*cII and *Bam*HI to obtain a 5.4 kb fragment containing a tetracycline-resistance gene (Tobian *et al.*, 1984). Plasmid pPGS749 (Kuramitsu & Wang, 2006) was digested with *Sma*I and *Bgl*II, and a 2.2 kb fragment that contains a Rep origin which replicates in streptococci was purified using a QIAEX II Gel Extraction kit (Qiagen). The two fragments were ligated using T4 ligase to generate pTet, a shuttle plasmid with tetracycline resistance that replicates in both *E. coli* and streptococci. pTet was then used for complementation of the *arcA* gene. The encoding region of CC5A *arcA* along with 330 bp of upstream sequence from the potential start codon was amplified by PCR with primers 5′-GCGGTACCTCAGCTATGAGCACAAACAG (*Kpn*I site underlined), and 5′-GCCCATGGACAACGAGGTCCACCACG (*Nco*I site underlined). The PCR product was cloned into pTet vector. The recombinant plasmid, pT-ARCA, was introduced by transformation into the *arcA*-deficient mutant, *S. cristatus* ArcAE, to create *S. cristatus* cArcAE. After transformation, erythromycin- and tetracycline-resistant transconjugants were selected, and plasmid identity was confirmed by PCR analysis.

### Cloning and expression of the *arcA* gene in *E. coli*.

*arcA*, encoding arginine deiminase, was amplified by PCR with primers 5′-GCGGTACCTATGTCTACACATCCAATTC-3′ (*Kpn*I site underlined) and 5′-GCGAGCTCACAACGAGGTCCACCACG-3′ (*Sac*I site underlined), which produced a 1200 bp PCR product. The PCR product was then cloned into pCRII-TOPO (Invitrogen). Recombinant arginine deiminase (rArcA) was expressed in *E. coli* by using a pThiohis protein expression system (Invitrogen). The *arcA* DNA fragment was subcloned into pThiohis-A downstream of a His tag. The recombinant ArcA was expressed in *E. coli* DH5*α* cells carrying the pThiohis-A/arcA plasmid in the presence of IPTG and kanamycin. His-tagged rArcA was purified with ProBond resin (Invitrogen). The His-tag on the recombinant protein was cleaved with enterokinase and removed by His-bind resin. Enterokinase was then removed by using Ekapture agarose.

### Arginine deiminase assay.

The arginine deiminase assay was performed in 96-well microplates as described by Thirkill *et al.* (1983). *S. cristatus* CC5A protein samples were adjusted with PBS to a constant 100 μl volume in each well, and mixed with 50 μl 0.1 M l-arginine. The mixtures were allowed to react for 1 h at 37 °C and the reactions were then terminated by the addition of 50 μl 20 % sulfuric acid. Finally, 1 % 2,3-butanedione monoxime (Sigma) was added to each well, and the reaction was developed by incubation in the dark for 1 h at 56 °C. The peach colour was quantified with a Benchmark plus microplate spectrophotometer (Bio-Rad) at 492 nm.

### Construction of *P. gingivalis* Mflac strain.

A *P. gingivalis* strain carrying an *mfa1* promoter–*lacZ* fusion was generated by the method described before (Xie *et al.*, 1997). Briefly, the *mfa1* promoter region was amplified by PCR with primers 5′-ACCCATCCTCTGTCTTCTGC-3′ and 5′-CTCGTTATCACATATCCGAACC-3′, and cloned into pDN19lac to generate the *mfa1* promoter–*lacZ* fusion. The recombinant plasmid was introduced into *P. gingivalis* ATCC 33277 by conjugation. The *P. gingivalis* transconjugants (Mflac) were selected on TSB plates containing 10 μg erythromycin ml^−1^.

### *β*-Galactosidase assays.

*S. cristatus* protein fractions (25 μg) were mixed with 10^5^ cells of *P. gingivalis* UPF, which contains a chromosomal *fimA* promoter–*lacZ* reporter construct, and spotted onto a TSB blood agar plate. The ability of the fractions to inhibit *fimA* expression in *P. gingivalis* was determined with a *β*-galactosidase assay. Expression of the *lacZ* gene under control of the *fim*A promoter was measured by the standard spectrophotometric *β*-galactosidase assay with ONPG as the substrate, as described by Xie *et al.* (1997).

## RESULTS

### Identification of *S. cristatus* inhibitory protein

**Fig. 1. f1:**
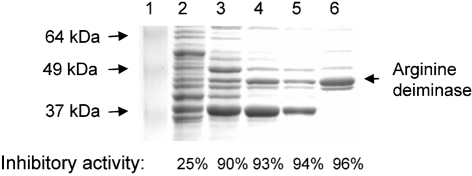
SDS-PAGE analysis and inhibitory activity of the fractions of *S. cristatus* surface proteins. Surface extracts of *S. cristatus* were precipitated with ammonium sulfate at increasing concentrations, separated by SDS-PAGE and stained with Coomassie blue. Lane 1, molecular size standards; lane 2, ammonium sulfate (AS) fraction AS3; lane 3, AS4; lane 4, AS5; lane 5, AS6; lane 6, Blue Sepharose unbound fraction of AS6. Molecular sizes and the ArcA band are denoted by arrows. Inhibitory activity is expressed as % reduction (compared to buffer control) of LacZ activity in *P. gingivalis* UPF, a strain carrying the transcriptional fusion of a promoterless *lacZ* and the *fimA* promoter region.

We reported previously that the expression of the *fimA* gene is repressed in the presence of surface extracts of *S. cristatus*, but not in the culture medium, indicating the presence of a LuxS-independent intergeneric communication system (Xie *et al.*, 2000). To purify the signal molecule, we first fractionated *S. cristatus* CC5A surface extracts by ammonium sulfate precipitation (Xie *et al.*, 2004). For further purification, the active fraction (AS6, 1 ml) was then applied to a Blue Sepharose column to remove glyceraldehyde-3-phosphate dehydrogenase, one of the major proteins in the AS6 fraction. The non-bound proteins were collected from the column and the fractions were analysed by SDS-PAGE. To test their ability to repress *fimA* expression in *P. gingivalis*, each fraction was mixed with *P. gingivalis* UPF, a strain carrying a *fimA* promoter–*lacZ* fusion. Expression of the *lacZ* gene under the control of the *fimA* promoter was measured by *β*-galactosidase assay (Xie *et al.*, 1997). The results shown in Fig. 1 reveal that a protein band of approximately 47 kDa had a high correlation with the inhibitory activity. The ability to repress *fimA* expression was enhanced as the purity of this 47 kDa protein increased. The expression of *fimA* in *P. gingivalis* was inhibited by as much as 96 % by the unbound fraction after Blue Sepharose column chromatography (Fig. 1). These data suggested the involvement of the 47 kDa protein in intergeneric communication between *S. cristatus* and *P. gingivalis.*

To identify the 47 kDa protein, we performed in-gel digestion followed by MALDI-TOF mass spectrometry, as described in Methods. Searches against the SWISS-PROT database identified the protein as arginine deiminase (ArcA). Identification was accepted based on the significant molecular weight search (MOWSE) scores. Protein scores greater than 66 are significant (*P*<0.05). The score for the 47 kDa protein was 604. The molecular mass (46 752 Da) of streptococcal ArcA is consistent with the corresponding region of the gel as determined by the molecular mass markers.

### Activity of the *arcA* mutant and complemented strains

**Fig. 2. f2:**
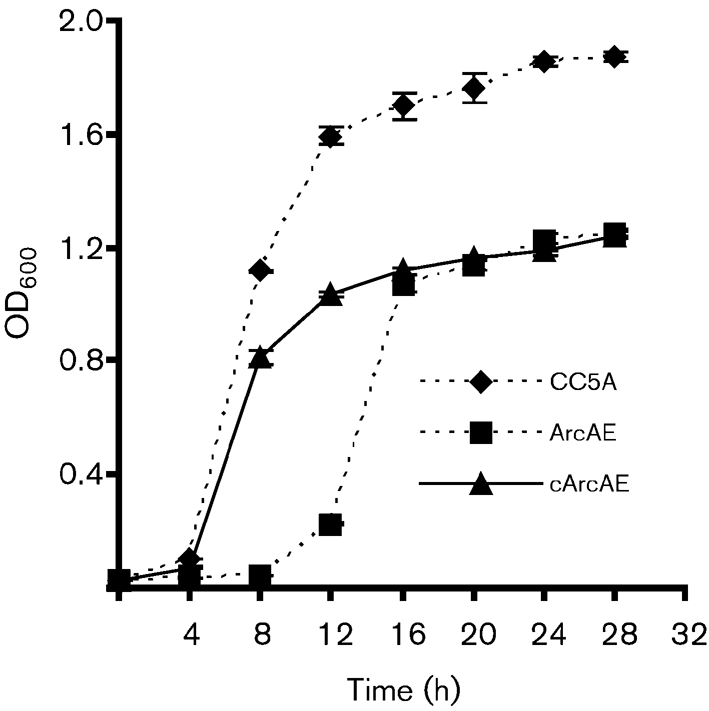
Comparison of the growth curves of *S. cristatus* strains in TSB medium. The data points are means±sem of four samples (error bars not shown where smaller than symbols). Samples of 1 ml were taken and the OD_600_ was measured over a period of 30 h.

**Fig. 3. f3:**
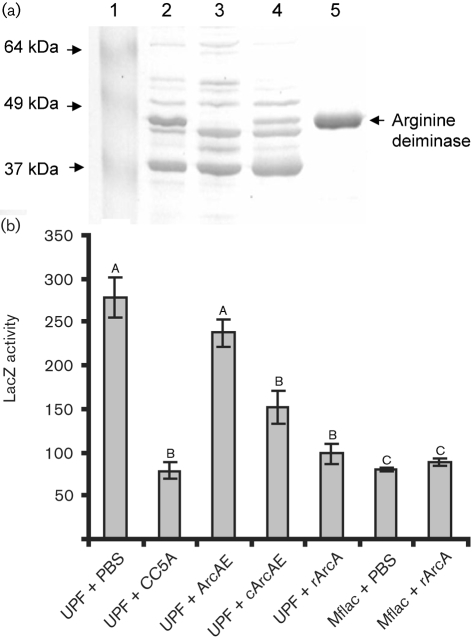
Inhibition of *fimA* expression in *P. gingivalis* by ArcA. (a) *S. cristatus* surface proteins were subjected to SDS-PAGE (12 %) and stained with Coomassie blue. Lane 1, molecular mass markers; lane 2, ammonium sulfate fraction AS6 of CC5A; lane 3, ammonium sulfate fraction AS6 of ArcAE (*arcA* mutant); lane 4, ammonium sulfate fraction AS6 of cArcAE (*arcA* mutant complemented with the wild-type allele); lane 5, recombinant ArcA purified from *E. coli*. (b) *P. gingivalis* UPF carrying a *fimA* promoter–*lacZ* fusion and *P. gingivalis* Mflac carrying an *mfa1* promoter–*lacZ* fusion were tested for LacZ activity in the presence or absence of surface extracts (50 μg) isolated from the *S. cristatus* strains indicated, or rArcA. The results are means±sem (*n*=3). Means with different letters are significantly different (*P*<0.05; Bonferroni test); means with the same letter are not significantly different.

To confirm the role of ArcA in regulation of *fimA* expression, we constructed an *arcA* mutant of *S. cristatus*. Insertional inactivation of the *S. cristatus arcA* gene resulted in a prolonged lag period under the standard growth conditions for streptococci (Fig. 2). This is not surprising since the arginine deiminase pathway is partly responsible for ATP regeneration in bacteria (Crow & Thomas, 1982). Comparison of the ammonium sulfate precipitation fractions AS6 between wild-type CC5A and the mutant strain ArcAE showed that a 47 kDa band was missing from the mutant (Fig. 3a[Fig f3]). Furthermore, mutation of *arcA* abrogated the inhibitory activity toward *P. gingivalis fimA* expression (Fig. 3b[Fig f3]), indicating that arginine deiminase is indeed an effector molecule mediating communication between *S. cristatus* and *P. gingivalis*.

The arginine deiminase operon has been extensively studied in *S. gordonii* DL1 (Caldelari *et al.*, 2000; Dong *et al.*, 2002; Zeng *et al.*, 2006) and consists of five genes that encode enzymes involved in the conversion of arginine to ornithine, ammonia and CO_2_ with the concomitant production of ATP (Dong *et al.*, 2002). *arcA* is the first gene in this operon. To eliminate the possibility that a polar effect plays a role in abolishing inhibitory activity in the *arcA* mutant, we complemented the mutant with the wild-type allele *in trans*. As shown in Fig. 3(a), production of ArcA was restored in the complemented strain cArcAE, although the expression level was lower compared to the parental CC5A strain. Complementation of the *arcA* mutant with the *arcA* gene partially restored the wild-type phenotype, since surface extracts isolated from the complemented strain cArcAE inhibited 50 % of *fimA* expression in *P. gingivalis* (Fig. 3b[Fig f3]).

### Activity of recombinant ArcA protein

We further confirmed the role of arginine deiminase in the repression of *fimA* expression in *P. gingivalis* by cloning and expressing *arcA* in *E. coli*. The *fimA* expression was repressed 2.5- to 3-fold in the presence of the recombinant protein (rArcA) (Fig. 3b[Fig f3]), although the inhibitory activity was not as high as that of the natural protein, which was able to inhibit 96 % of the *fimA* expression (Fig. 1). This could be due to incorrect folding or post-translational modification in the heterologous host. The role of rArcA in expression of the short fimbriae (*mfa1*) was also examined by using a *P. gingivalis* strain carrying an *mfa1–lacZ* fusion. In the presence of rArcA, the promoter activity of *mfa1* was not modulated in *P. gingivalis* (Fig. 3b[Fig f3]), suggesting a specific role of *S. cristatus* ArcA in *fimA* expression. As a control, a major surface protein, glyceraldehyde-3-phosphate dehydrogenase (GAPDH), of *S. cristatus* CC5A was also cloned and expressed in *E. coli*. The rGAPDH had no effect on *fimA* expression (data not shown).

### Dual function of arginine deiminase

While the arginine deiminase system is found in many bacteria (Burne & Marquis, 2000), relatively few arginine deiminase-positive bacteria are found in oral biofilms (Zeng *et al.*, 2006). Arginine deiminase catalyses the hydrolysis of l-arginine to l-citrulline and ammonia, and the latter is believed to be important for oral biofilm pH homeostasis and caries prevention (Burne & Marquis, 2000). Besides arginase activity, ArcA can also function as an inhibitor of angiogenesis and tumour growth, which may be due to the depletion of arginine (Gong *et al.*, 2000; Kang *et al.*, 2000; Park *et al.*, 2003). In addition, arginine deiminase plays an important role in the regulation of the level of nitric oxide that is synthesized by NO synthase from arginine, a substrate of arginine deiminase (Gotoh & Mori, 1999). Since these two enzymes compete for the same substrate, antiangiogenic activity may result from the suppression of nitric oxide generation. To address whether the inhibitory activity of ArcA depends on enzyme activity, we examined each fraction for its arginase activity. Relatively high arginine hydrolytic activity was detected in the surface extract of *S. cristatus* (Table 2). Arginine hydrolytic activity was abolished in the *arcA* mutant, but was partially restored in the surface extracts of the *arcA*-complemented strain, which is consistent with production of arginine deiminase. Surprisingly, the purified fraction of arginine deiminase (the unbound fraction of the Blue Sepharose column) did not show an increased hydrolytic activity, despite the fact that at least 10 times more inhibitory activity was found in the purified fraction than in the surface extracts (Table 2). We speculated that the arginase activity is not required for intergeneric communication between *S. cristatus* and *P. gingivalis*. To test this hypothesis, communication was tested in the presence of aminoguanidine (20 μM) and l-lysine (5 mM), both of which are arginine deiminase inhibitors (Ulisse *et al.*, 2001). These agents completely inhibited the arginase activity in CC5A fractions, but had little effect on the inhibitory activity of the fractions on *fimA* expression in *P. gingivalis* (Table 2). These data suggest that the catalytic activity of ArcA is not required for the mechanism of inhibition of *fimA* expression. It appears that ArcA now joins a growing list of bacterial proteins that can have multiple functions, possibly depending on their location (Jeffery, 1999).

## DISCUSSION

*P. gingivalis* is a secondary colonizer of dental plaque, and is significantly more prevalent in both supra- and subgingival plaque samples from periodontitis subjects in comparison with healthy subjects (Ximenez-Fyvie *et al.*, 2000). The surface attachment of *P. gingivalis* is promoted by adhesive molecules including fimbriae. The long fimbriae, composed of the FimA subunit, mediate adherence of *P. gingivalis* to a variety of oral substrates and molecules, including proline-rich proteins and glycoproteins, statherin, fibrinogen, fibronectin and lactoferrin (Lamont & Jenkinson, 1998). The fimbriae are also important effector molecules in coaggregation interaction with various early plaque-forming bacteria, such as *Actinomyces viscosus* (Goulbourne & Ellen, 1991), *Streptococcus gordonii* (Lamont *et al.*, 1993) and *Streptococcus oralis*. Amano *et al.* (1997) also demonstrated that the FimA C-terminal region is involved in coaggregation with *S. oralis*, with functional domains located in regions spanning amino acids 266–286 and 287–337. FimA is also a specific adhesin mediating coaggregation of *P. gingivalis* and *Treponema denticola*, another secondary colonizer (Hashimoto *et al.*, 2003). This specific coaggregation ability with other oral bacteria suggests that the *P. gingivalis* long fimbriae contribute to bacterial integration into dental plaque by interacting with the early and secondary colonizers of dental plaque. Evidently, dental plaque colonization is beneficial to *P. gingivalis* survival in their optimum ecological niche, periodontal pockets. Our earlier finding demonstrated that *S. cristatus* is able to repress expression of the *fimA* gene in *P. gingivalis* and thus prevent subsequent heterotypic biofilm formation (Xie *et al.*, 2000). The present results provide evidence for the first time that the surface protein arginine deiminase of *S. cristatus* is a signal molecule responsible for cell–cell communication between *S. cristatus* and *P. gingivalis*. As a result of this signal, *P. gingivalis* shuts down expression of the *fimA* gene. Communication between Gram-positive and Gram-negative bacteria as observed here may be fundamental to bacteria in multispecies biofilms. Interspecies cooperation and competition play important roles in biofilm development and organization. The identification of the molecular basis for an intergeneric contact-dependent communication system provides a molecular basis to begin to understand the differentiation of oral microbial communities from commensal to pathogenic. The study presented here could ultimately lead to the development of novel means to inhibit oral colonization of periodontal pathogens. Oral streptococci are some of the predominant early colonizers of oral plaque (Li *et al.*, 2004), and this unique communication system of sensing foreign species via a surface protein may have been developed to uphold a dominant position in this specialized niche. The consequent inhibition of *P. gingivalis* biofilm formation suggests that susceptibility to periodontal disease may depend to some extent on the microbial composition of the early plaque biofilm and, moreover, that production of arginine deiminase by the oral streptococci may be significant in protection against periodontitis.

## Figures and Tables

**Table 1. t1:** Strains and plasmids used in this study

**Strain or plasmid**	Relevant characteristics*	**Source or reference**
***S. cristatus***		
CC5A	Low-passage plaque isolate	Lab collection
ArcAE	Derivative of CC5A containing an insertional mutation in the *arcA* gene; Em^r^	This study
cArcAE	A complemented strain of ARCE harbouring pT-ARCA	This study
***P. gingivalis***		
ATCC 33277	Type strain from ATCC	Lab collection
UPF	Derivative of ATCC 33277 containing *fimA*–*lac*Z gene fusion in its chromosomal DNA; Em^r^	Xie *et al.* (1997)
Mflac	Derivative of ATCC 33277 containing *mfa1*–*lac*Z gene fusion in its chromosomal DNA; Em^r^	This study
***E. coli***		
DH5*α*	F^−^*φ*80d*lac*ZΔ(*lacZYA–argF*)U169 *endA1 supE44recA1 relA1*	BRL
**Plasmids**		
pVA3000	A suicide vector for *Bacteroides*; Em^r^, 5.3 kb	Lee *et al.* (1996)
pDN19lac	Contains a promoterless *lacZ* gene	Xie *et al.* (1997)
pJRD215	A wide-host-range plasmid	Xie *et al.* (1997)
pPGS749	*E. coli–Streptococcus* shuttle plasmid with Em^r^	Kuramitsu & Wang (2006)
pSF143	Suicide vector for streptococci with Tet^r^; replicates only in *E. coli*	Tao *et al.* (1992)
pTet	Shuttle plasmid derived from both pPGS749 and pSF143 with Tet^r^; replicates in both *E. coli* and streptococci	This study
pT-ARCA	pTet plasmid carrying the *arcA* gene of *S. cristatus* CC5A	This study
pCRII-TOTO	A linearized plasmid with single 3′ dT residues; Km^r^ Am^r^	Invitrogen

*Km^r^, Tet^r^, Em^r^, Am^r^, resistance to kanamycin, tetracyline, erythromycin and ampicillin, respectively.

**Table 2. t2:** Comparison of arginase activity and the inhibitory activity in protein fractions of *S. cristatus*

**Protein fraction**	Arginine deiminase activity*	**LacZ acivity†**
PBS	0.04±0.00	278±21
*S. cristatus* CC5A surface extract (50 μg)	2.12±0.06	78±10
*S. cristatus* ArcAE surface extract (50 μg)	0.18±0.01	237±15
*S. cristatus* cArcAE surface extract (50 μg)	1.46±0.10	152±18
CC5A surface extract (50 μg)+10 mM aminoguanidine	0.32±0.04	nd
CC5A surface extract (50 μg)+5 mM lysine	0.14±0.01	nd
Purified fraction AS6 (25 μg)	0.8±0.10	12±2
Purified fraction AS6 (25 μg)+10 mM aminoguanidine	0.15±0.02	19±3
Purified fraction AS6 (25 μg)+5 mM lysine	0.15±0.01	21±4

*Arginine deiminase levels are means±sd (*n*=3). †Expression of the *fimA* gene was determined by measuring LacZ activity, expressed in Miller units. Results are means±SEM (*n*=3). nd, Not determined.
